# Complicated trabeculectomy converted into *ab externo* cyclodialysis

**DOI:** 10.5935/0004-2749.2022-0046

**Published:** 2024-02-23

**Authors:** Daiane Beutinger, Candice Carolina de Mesquita Costa, Niro Kasahara

**Affiliations:** 1 Irmandade da Santa Casa de Misericórdia de São Paulo, São Paulo, SP, Brazil; 2 Faculdade de Ciências Médicas, Santa Casa de São Paulo, São Paulo, SP, Brazil

**Keywords:** Glaucoma/surgery, Trabeculectomy, Ophthalmologic surgical procedures /adverse effects, Cyclodialisys, Postoperative complications

## Abstract

The creation of a scleral flap during trabeculectomy can be complicated by a buttonhole, partial amputation at the limbus, and extensive thinning. In some cases, the procedure must be aborted to prevent more serious postoperative complications. This report describes a technique of converting complicated trabeculectomy into ab externo cyclodialysis. A 41-year-old patient with congenital glaucoma presented with a perforated scleral wall with the choroidal tissue exposed during the dissection of the partial-thickness scleral flap. By using a Barraquer cyclodialysis spatula through the scleral perforation, the choroid was separated from the sclera up to the scleral spur over 30° into the anterior chamber. The sclera and conjunctiva/Tenon were sutured with 10-0 nylon single sutures. Two months later, the intraocular pressure was reduced to 16 mmHg with no hypotensive topical medications. This case illustrates an alternative approach to managing a flap-related perioperative complication in trabeculectomy, which yielded good early results.

## INTRODUCTION

Trabeculectomy is the procedure of choice for the surgical management of glaucoma^(1)^. It involves the crea-tion of a scleral flap that covers an inner sclerostomy by which the aqueous humor is drained through the subconjunctival space, creating a bleb. Despite being a safe procedure, perioperative complications can occur, such as transient anterior chamber bleeding, subconjunctival hemorrhages, conjunctival buttonholes, partial transection of the superior rectus tendon, serous choroidal detachments, and scleral flap problems, e.g., buttonholes, partial amputation at the limbus, and extensive thinning^(2)^. These complications must be managed accordingly.

We recently confronted an intraoperative complication in trabeculectomy, which prompted us to report its management and outcomes.

## CASE REPORT

**Figure 1:**
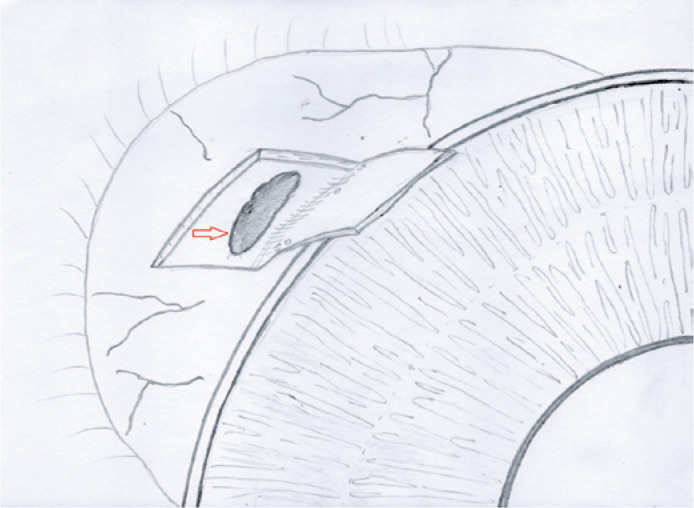
Choroidal tissue exposed under the scleral flap (arrow).

A 41-year-old female patient had previously undergone multiple procedures for congenital glaucoma in her right eye. She was taking four hypotensive topical medications, namely, timolol maleate 0.5%, brimonidine 0.2%, dorzolamide 2%, and bimatoprost 0.03%. Her uncorrected visual acuity was 20/20 in the right eye (OD) and 20/30 in the left eye (OS). On biomicroscopy, both eyes had megalocornea, the anterior chamber was deep and quiet, and both lenses were clear. The intraocular pressure (IOP) was 27 mmHg OD and 21 mmHg OS, and both optic discs had concentric enlargement of the cup. The cup-to-disc ratio was 0.9 OD and 0.8 OS. Automated perimetry revealed a central island OD (MD -18.7 dB) and a superior arcuate scotoma OS (MD -10.2). Since she was considered to have an advanced disease and needed a low target pressure, trabeculectomy was performed in her right eye. During surgery, the sclera was very thin; when dissecting the flap, we inadvertently perforated the scleral wall and the choroidal tissue herniated (Figure 1). We continued dissecting the scleral flap beyond that point; however, the flap was very thin and friable.

At this point, we had a few options: to abort the procedure completely, create another flap using donor scleral tissue and suture it to replace the original on, or continue with the planned surgery with possible diffi-culty in suturing the scleral flap and the prospects of postoperative overfiltration and hypotony.

**Figure 2:**
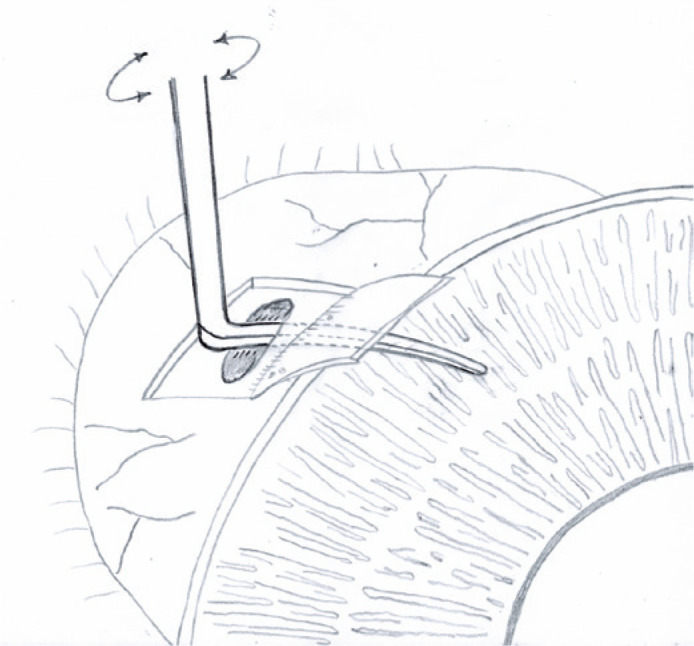
A Barraquer cyclodialysis spatula was inserted through the inadvertent scleral perforation and was moved toward the anterior chamber to separate the choroid from the sclera.

Given the unavailability of donor tissue, another approach was chosen: convert such trabeculectomy into an *ab externo* cyclodialysis. A Barraquer cyclodialisys spatula (Storz^®^ Ophthalmic Instruments, Heidelberg, Germany) was introduced in the scleral perforation toward the anterior chamber to separate the choroid from the sclera up to the scleral spur over 30° (Figure 2). The scleral perforation was then sutured with two 10-0 nylon sutures, and the flap was sutured with the other two watertight sutures. The conjunctiva and Tenon’s membrane were sutured with two wing 10-0 nylon sutures. On postoperative day 1, the IOP was 10 mmHg, the anterior chamber was deep with a few red blood cells, and a superior bleb was noticeable. On gonioscopy, a small cyclodialysis cleft was evident in the superior quadrant of the iridocorneal angle. Two months later, the bleb was still present, and the IOP was 16 mmHg.

## DISCUSSION

Before the development of trabeculectomy, cyclodialysis was a surgical option for the treatment of glaucoma^(3,4)^. The surgical technique involved separating the longitudinal ciliary muscle fibers from the scleral spur under direct gonioscopic view (*ab interno*) and creating an alternative pathway for aqueous humor drainage; however, the procedure could be complicated by ocular hypotony^(5)^. Sihota et al. described a variation in the original technique, i.e., *ab externo* cyclodialysis, to enhance trabeculetomy in patients with post-penetrating keratoplasty glaucoma. After the dissection of a partial--thickness rectangular scleral flap and mitomycin-C application, the scleral spur was identified, and a 3-mm long, circumferential, deeper corneoscleral block - 1 mm in front and 1 mm behind the scleral spur - was excised^(6)^. Different from our technique, the author did not use a Barraquer spatula to separate the choroid from the sclera up to the scleral spur and the anterior chamber.

Long-term results of the combined trabeculectomy--cyclodialysis surgery indicated that the majority of patients needed additional topical medication for IOP control^(7)^. As the latest development, Dada et al. used Ologen implant in three sites during trabeculectomy-cyclodialysis surgery with encouraging short-term IOP control^(8)^.

In this case, the IOP was probably reduced by both uveoscleral outflow and external filtration, as evidenced by a superior conjunctival bleb. Aqueous humor was probably draining from the scleral flap after going its way from the anterior chamber to the inadvertent scleral perforation. This was not the original purpose because we planned on a watertight suture of the scleral flap. The flap was probably very thin, and the sutures were not enough to provide an impermeable scleral wound. However, we believe this was favorable for IOP control.

Potential complications of the original cyclodialysis include hypotony and hemorrhage^(3)^. In patients with post-penetrating keratoplasty glaucoma who underwent ab externo cyclodialysis, one patient experienced endothelial rejection 2 years later. Other potential complications such as graft failure, severe hypotony, or suprachoroidal bleeding have not been reported^(4)^. In our patient, early hypotony and suprachoroidal hemorrhage were not observed.

Unfortunately, we had no long-term results of the patient’s treatment. We are unsure of how long the bleb would still be functioning, even if mitomycin-C (0.2 mg/mL) was applied - this was a young patient with increased chances of scarring. We do not know if the uveoscleral flow by the cyclodialysis cleft would be sufficient for the drainage of aqueous humor for good IOP control. In addition, an ultrasound biomicroscopy image to ensure the presence of a suprachoroidal cleft was not obtained, although the gonioscopy revealed that a small cyclodialysis cleft was evident in the superior quadrant of the iridocorneal angle.

In summary, we described an optional approach to convert complicated trabeculectomy into an alternative procedure that has yielded relatively good short-term IOP control.
